# Dry eye disease caused by viral infection: Past, present and future

**DOI:** 10.1080/21505594.2023.2289779

**Published:** 2023-12-04

**Authors:** Min Wu, Cuilian Sun, Qin Shi, Yalu Luo, Ziyu Wang, Jianxiang Wang, Yun Qin, Weihang Cui, Chufeng Yan, Huangyi Dai, Zhiyang Wang, Jia Zeng, Yamei Zhou, Manhui Zhu, Xiaojuan Liu

**Affiliations:** a Department of Pathogen Biology, Medical College, Nantong University, Nantong, Jiangsu, China; b Department of General Medicine, Gongli Hospital, Shanghai, China; c Suzhou Medical College, Soochow University, Suzhou, Jiangsu, China; d Medical College, Nantong University, Nantong, Jiangsu, China; e Department of Microbiology Laboratory, Jiaxing Center for Disease Control and Prevention, Jiaxing, Zhejiang, China; f Department of Ophthalmology, Lixiang Eye Hospital of Soochow University, Suzhou, Jiangsu, China

**Keywords:** Dry eye disease (DED), lacrimal gland (LG), viral infection, inflammation, corneal epithelial cells (CECs)

## Abstract

Following viral infection, the innate immune system senses viral products, such as viral nucleic acids, to activate innate defence pathways, leading to inflammation and apoptosis, control of cell proliferation, and consequently, threat to the whole body. The ocular surface is exposed to the external environment and extremely vulnerable to viral infection. Several studies have revealed that viral infection can induce inflammation of the ocular surface and reduce tear secretion of the lacrimal gland (LG), consequently triggering ocular morphological and functional changes and resulting in dry eye disease (DED). Understanding the mechanisms of DED caused by viral infection and its potential therapeutic strategies are crucial for clinical interventional advances in DED. This review summarizes the roles of viral infection in the pathogenesis of DED, applicable diagnostic and therapeutic strategies, and potential regions of future studies.

## Introduction

The clinical manifestations of a DED patient are tear volume reduction, ocular surface inflammation and damage, and high osmotic pressure of the tear film [1]. In 2017, the Tear Film and Ocular Surface Society’s Dry Eye Workshop II (TFOS DEWSII) provided a new definition of DED based on the new findings, declaring that DED is a multifactorial disease of the ocular surface characterized by a loss of homoeostasis of the tear film, accompanied by ocular symptoms, in which tear film instability and hyperosmolarity, ocular surface inflammation and damage, as well as neurosensory abnormalities play aetiological roles [2]. DED is widely prevalent and multifactorial in nature. Epidemiological studies explore the prevalence of DED depending on the definition and diagnosis of the disease, and the population surveyed. In Netherlands, a cross-sectional study including 79,866 participants between 2014 and 2017 reveals a 9.1% prevalence of DED. Female and older individuals are more likely to be affected by DED [3]. Inflammation, electronic products, diabetes mellitus, laser-assisted in situ keratomileusis (LASIK), too little and poor sleep quality, and sex hormones contribute to DED [4–7]. Ocular surface disease index (OSDI) or dry eye questionnaire (DEQ-5) are used to evaluate patients’ subjective symptoms. Tear film stability is usually evaluated by tear break-up time (TBUT). Tear film composition is evaluated by tear film osmolarity. Ocular surface staining is used to detect the damage to ocular surface. If the patients DEQ ≥ 6 or OSDI ≥ 13 with one of other diagnostic results including TBUT < 10 or osmolarity ≥ 308mosm/L/interocular difference > 8 mosm/L or ocular surface staining > 5 corneal spots, > 9 conjunctival spots/lid margin (≥2 mm length or ≥ 25% width) are diagnosed with DED [8]. Meanwhile, different factors cause different types of DED. The subtype classification of DED is evaluated by the severity of meibomian gland dysfunction (MGD) feature (Meibum quality, symptoms, and corneal staining) [9], lipid thickness/dynamics, and tear volume assessment [8]. In 2017, TFOS DEWS II classified DED into three types: aqueous-deficient, evaporative, and mixed [2].

Aqueous-deficient dry eye (ADDE) always manifests as tear volume reduction combined with lacrimal gland (LG) dysfunction and inflammation. Sjogren’s syndrome is a chronic systemic autoimmune disease, which is a form of ADDE characterized by lymphocytic infiltration of exocrine glands (lacrimal glands, abbreviated to LGs). Patients with evaporative dry eye (EDE) often show increased evaporation of the aqueous tear phase from the exposed ocular surface. Meibomian gland dysfunction (MGD) contributes to EDE. Patients with MGD display abnormal lipid biosynthesis and meibomian gland (MG) duct obstruction. Additionally, the combination of ADDE and EDE leads to tear hyperosmolarity, finally resulting in inflammation, cell death, and loss of conjunctival goblet cells. Therefore, besides ADDE and EDE, a proportion of patients are diagnosed with mixed dry eye (MDE) [10–12].

During viral infection, the host innate immune system senses viral products, such as viral nucleic acids, to activate innate defence pathways, promote inflammation and apoptosis, and reduce cell proliferation [13]. Viruses, such as severe acute respiratory syndrome coronavirus 2 (SARS-CoV-2), human immunodeficiency virus (HIV), hepatitis B virus (HBV), hepatitis C virus (HCV), Epstein-Barr virus (EBV), and human T-cell lymphotropic virus-1 (HTLV-1) [14], induce inflammation and sicca syndrome, consequently triggering morphological and functional changes on the LG and ocular surface.

This review summarizes the roles and mechanisms of viral infection in the pathogenesis of DED, applicable mechanisms, and implications for diagnostic and therapeutic strategies. Finally, we shed new light on the potential paths for future studies of viral infection-associated DED.

### SARS-CoV-2

Coronavirus disease 2019 (COVID-19) is a pandemic caused by SARS-CoV-2 [15]. SARS-CoV-2 infection activates both the innate and adaptive immune responses. In the later phase, infection of mononuclear cells induces a massive inflammatory response, which leads to local and systemic tissue damage. For patients with severe COVID-19, the numbers of CD4^+^ T cells, CD8^+^ T cells, B cells, and natural killer (NK) cells are decreased in the whole body [16–18]. Thus, COVID-19 complications include acute liver, cardiac, and kidney injuries, as well as inflammatory responses and secondary infections [16].

As of SARS-CoV-2 is transmitted by aerosols and/or droplets [19], the widespread use of face mask-caused DED has become more common [20,21]. Meanwhile, the increase in discomfort symptoms in the ocular region is mostly part of DED, resulting in a new term called mask-associated dry eye (MADE) [22]. Recently, a questionnaire was administered to 6925 Chinese participants from 29 January 2021, to 8 February 2021, among which 5973 participants wore face masks for ≥6 months. The results revealed that 7.90% of the participants had MADE. Subjects who had continuous (approximately 8 h a day for over 6 months) wearing face masks showed an increased OSDI and decreased TBUT, accompanied by increased T cells, leukocytes, and natural killer T (NKT) cells on the ocular surface [23]. These data suggest that wearing masks may amplify the immune response on the ocular surface. During the ongoing COVID-19 pandemics, outdoor activities and social engagement have been reduced, while indoor activities, which are mainly attributed to screen time (time viewing screens of computers), have increased [24,25]. A longer blinking interval exacerbates the evaporation of tears and, consequently, increases the risk of developing DED. It has been reported that a long screen time aggravates dry eye symptoms, especially in patients with moderate DED [26].

The prevalence of COVID-19 has increased the number of DED patients. A recent study conducted a survey to USA COVID-19 patients who self-reported DED. In this survey, a large proportion (61%) of patients were diagnosed with Sjögren’s syndrome [26]. A prospective study used different social media platforms to collect data on participants’ age, sex, profession and DED symptoms. These results confirm that a large proportion of COVID-19 patients suffer from DED. Reverse transcription polymerase chain reaction (RT-PCR) results show that SARS-CoV-2 exists in conjunctival secretions and tears of patients with conjunctivitis [27]. In COVID-19 patients who have discomfort symptoms in the ocular region, tear film break-up times are decreased. Conjunctival impression cytology (CIC) results indicate that SARS-CoV-2 infection causes an alteration to ocular the surface and conjunctiva featured by decreased number and size of conjunctival goblet cells and morphological changes in corneal epithelial cells (CECs). In addition, neutrophils, which induce conjunctival inflammation and squamous metaplasia in the conjunctiva, increase the ocular surface [28].

Viral infection may be transmitted through the eye route [29], suggesting that the virus may enter the ocular surface to infect the eye. Studies have shown that SARS-CoV-2-associated proteins, including angiotensin-converting enzyme 2 (ACE2), transmembrane protease serine type 2 (TMPRSS2), and basigin (CD147), are expressed on human ocular surface. ACE2 acts as a SARS-CoV-2 receptor, mediating adherence of SARS-CoV-2 to host cells [30]. TMPRSS2 allows the fusion of SARS-CoV-2 and cellular membranes, enabling the entry of SARS-CoV-2 into the host cells [31]. Single-cell RNA sequencing (scRNA-seq) datasets show that ACE2 and TMPRSS2 are co-expressed by the limbal and conjunctival epithelium at the highest level (6.6% of the cells) [32]. CD147 is a transmembrane glycoprotein of the immunoglobulin superfamily that participates in bacterial and viral infection [33]. Wang et al. applied surface plasmon resonance (SPR), enzyme-linked immunosorbent assay (ELISA), and co-immunoprecipitation (Co-IP), verifying that CD147 interacts with the SARS-CoV-2 spike protein. SARS-CoV-2 enters host cells via CD147-mediated endocytosis [34]. CD147 is detected in human conjunctiva, cornea, and ocular fluids [35,36].

Moreover, SARS-CoV-2 single guide RNA (sgRNA) has been found in COVID-19 the cornea, limbus, sclera, and retinal pigment epithelium cells [37,38]. SARS-CoV-2 infects the limbal and corneal cells via nuclear factor kappa B (NF-κB) [39]. The signalling components RELB proto-oncogene, NF-κB subunit (RELB), interleukin-6 (IL-6), and chemokine C-X-C motif ligand 1 and 6 (CXCL1 and CXCL6), are expressed with the highest augment in SARS-CoV-2-infected corneas, limbus, and sclera following NF-κB activation [38].

Increased electronic screen time and poor quality of masks lead to impaired ocular surface function. Reduction in conjunctival goblet cells decreases tear production. Experts recommend that DED patients should to ensure that the mask is worn appropriately, apply lubricating drops, decrease the time in air-conditioned environments, and take regular breaks from digital devices to reduce screen time. Researchers are currently exploring potential defensive medicines against SARS-CoV-2. Ozone (O_3_) is used to disinfect and treat infectious diseases and inactivate viruses, fungi, and yeast in clinical practice [40]. Ozonated oil, a novel O_3_ derived compound, has been used to treat ocular pain, external ocular infections and inflammation. It can be stabilized for topical use by creating ozonide through a reaction between ozone and the double bonds of a monounsaturated fatty acid [40]. Recently, a study reports that topical application of ozonated oil in liposome eye drop gels [31] is able to reduce SARS-CoV-2 infection on the ocular surface [41]. Topical use of OED can ameliorate DED pathologic changes, including reduction of corneal epithelium thickness, expression of matrix metalloproteinase 9 (MMP-9), and pro-inflammatory factor interleukin 8 (IL-8) on the ocular surface [41]. The proanthocyanidin (PAC) fraction in blueberry leaves has strong antiviral activity against HCV and HTLV-1 [42,43]. Moreover, PAC suppressed SARS-CoV-2 infection. BB-PAC fraction 7 (Fr7) can effectively inhibit the activity of ACE2 [44], reducing SARS-CoV-2 infection in the limbal and conjunctival epithelium.

### HIV

HIV reduces cell-mediated and humoral immunity, leading to a wide range of infections. It directly damages the brain and lungs via mononuclear cell infection and activation. Immune activation causes subtle systemic organ damage, such as hepatic and central nervous system diseases [45]. HIV primarily infects CD4^+^ T cells [46]. Neutrophils also play an important role in HIV infection and exhibit both host defence and pathological functions. HIV-1 single-stranded RNA40 (SSRNA40) activates neutrophils, causing them to release pro-inflammatory cytokines (TNF-α and IL-6) and produce reactive oxygen species (ROS) [47].

The structures and functions of exocrine glands, including salivary gland (SG) and LG, are damaged by HIV infection. The aetiology of DED in HIV patients is usually due to HIV-mediated lymphocytic infiltration in the LGs [48]. A clinical case study reported that the left LG mass of an HIV-infected patient shows dense inflammatory infiltration, composed of mature lymphocytes, plasma cells, and endothelial cells [49]. Chronic inflammation promotes cytokine secretion, resulting in LG dysfunction, which contributes to a reduction in tear production [50]. A cross-sectional study performed a battery of comprehensive eye assessments, including tear film osmolarity, extent of MG dropout, tear film stability, and ocular surface staining, in healthy controls and HIV-positive subjects. These results demonstrate that HIV infection induces extensive MG dropout and subsequent MGD [51]. DED causes changes in corneal nerve morphology and reduces the length of corneal nerves [52]. One study detected retinal nerve fibre layer thickness (RNFLT) and visual acuity in HIV-positive subjects with no clinically apparent ocular infection or other pathology, confirming that HIV infection leads to decreased RNFLT and low visual acuity [53]. Tear cytokines, such as epidermal growth factor (EGF) and interferon-inducible protein-10 (IP-10), are significantly elevated in HIV patients compared to those of normal subjects [54].

Combined antiretroviral therapy [48], defined as a combination of antiretroviral therapy with at least three drugs, dramatically decreases the prevalence of acquired immune deficiency syndrome (AIDS). With the widespread use of cART, the survival of HIV patients has been improved [55]. Accordingly, the prevalence of ocular findings in the HIV-infected population has been significantly reduced, as proven by the decreased number of opportunistic ophthalmic infections and blinding disorders [56]. This cross-sectional study treats HIV patients with cART for a minimum duration of 6 months. With cART, the mean CD4 count is reduced in DED patients with HIV infection [56].

### HTLV-1

HTLV-1 spreads worldwide, especially in Central Africa, South America, and Southwest of Japan [57]. The prevalence of HTLV-1 infection gradually increases with age, especially among the female. HTLV-1 is transmitted through three main pathways: mother-to-child, sexual, and contaminated blood products [57]. HTLV-1 infects T cells, B cells, and myeloid cell lineages, ultimately causing weakened immunity [58]. HTLV-1 infection is associated with ocular inflammatory diseases including conjunctivitis, Sjögren’s syndrome, interstitial keratitis, and polyneuropathies [59]. Additionally, HTLV-1 infection can induce eye inflammation and neoplastic infiltration [60].

In year 2009–2011, a cross-sectional study recruited 272 HTLV-1-infected individuals. Results shows that 21.7% of HTLV-1 patients presented with sicca syndrome. Pro-inflammatory cytokine TNF-α expression is higher in the peripheral blood mononuclear cells (PBMCs) of patients with sicca syndrome than in those without sicca syndrome [61]. The OSDI, TBUT test, Schirmer I test, and Rose Bengal staining were used to evaluate 96 HTLV-1-infected subjects’ DED symptoms, revealing that half of HTLV-infected subjects were diagnosed with DED [62]. A clinical measure targeting 129 HTLV-1-infected subjects showed that 44 (34.1%) subjects complained of dry mouth, whereas 18 (13.9%) had dry eye. The Schirmer’s test of only two subjects showed abnormal results. Eight subjects showed hyposalivation [63]. These studies suggest that HTLV-1 changes correspond to the patient’s clinical manifestations, without damaging the structures and functions of the exocrine glands.

Meanwhile, a case report showed that fluorescein and lissamine green staining of an HTLV-1-infected patient showed a defect in the cornea and conjunctiva, whereas the Schirmer test indicated tear deficiency. Consequently, this HTLV-1-infected patient is diagnosed with aqueous tear-deficient keratoconjunctivitis sicca [64]. The average RNFLT for HTLV-1-infected patients is less than that for healthy individuals [65].

HTLV-1 infection activates CD4^+^ T cells, which produce inflammatory cytokines that cause ocular inflammation. Topical and/or oral corticosteroid treatment improves intraocular inflammation by inhibiting cytokine production in HTLV-1-infected CD4^+^ T cells [60]. A recent study indicates that tacrolimus was effective in resolving autoimmune manifestations in HTLV-1-related overlap syndrome (dermatomyositis/Sjögren’s syndrome) [66]. Similarly, allogeneic haematopoietic stem cell transplantation (HSCT) is considered the optimal curative therapy. Following allogeneic HSCT, eye complications display significant improvement with decreased HTLV-1 proviral load (PVL) [60]. Adalimumab, infliximab (IFX), and fully human monoclonal TNF-α antibodies are common strategies for the treatment of inflammatory diseases. Furthermore, ADA and IFX can’t exacerbate inflammatory cytokines and chemokines or increase PVL in HTLV-1-infected T cells, suggesting that ADA and IFX are safe for use in HTLV-1 infectious conditions safely [67,68].

### EBV

EBV belongs to the human herpes virus (HHVs) family, also known as human herpesvirus 4 (HHV4), which is a gamma-type HHV [69,70]. The course of EBV infection is determined by the virus load and immune system state of an individual, along with gene composition, other infection history, and environmental factors [71]. EBV is transmitted to recipients through saliva but rarely through semen or blood. Primary EBV infection causes infectious mononucleosis (IM), that typically manifests as fever, pharyngitis, lymphadenopathy, and fatigue [72]. EBV infection is associated with lymphoid and epithelial malignancies, including diffuse large B-cell lymphoma (DLBCL), Hodgkin lymphoma (HL), and various types of T/NK-cell lymphomas [73].

Likewise, EBV infection is related to Sjögren’s syndrome [74,75], which usually manifests as dysfunctional exocrine glands with keratoconjunctivitis sicca and xerostomia [76]. A recent seroepidemiological study determines that EBV is closely correlated with Sjögren’s syndrome [77]. Initially, EBV infects epithelial cells of the oropharynx, gains access to the underlying tissue, releases it from the oropharyngeal epithelium, and finally activates B cells [71]. In India, an EBV-infected person is diagnosed with acute DED, as evidenced by a significant reduction in tear meniscus height derived from slit lamp examination and easily peeled thick membranes from the tarsal surface in both eyes. Nevertheless, lubricants and topical steroids can’t improve tear production [78].

Patients with DED are characterized by increased tear film osmotic pressure and ocular surface inflammation, causing severe ocular surface damage. c-Jun N-terminal kinase (JNK), mitogen-activated protein kinase (MAPK), and NF-κB pathways mediate inflammation in CECs, leading to ocular surface damage [79]. EBV infects human corneal epithelial cells (HCECs) via NF-κB activation. One study ascertained that EBV infection increased the expression of NF-κB subunits, including p65 and p50/p52, in HCECs [80]. EBV induces increased Toll-like receptor 3 (TLR3) expression in HCECs. Receptor-interacting protein-1 (RIP-1) and tumour necrosis factor receptor-associated factor 6 (TRAF6), which are recruited by the protein toll/interleukin-1 receptor (TIR) domain-containing adaptor, are expressed at higher levels in HCECs/EBV in EBV-induced HCECs than in normal HCECs. These data suggest that both TLR3/Toll/IL-1 R domain-containing adaptor-inducing IFN-β (TRIF) and retinoic acid-inducible gene I (RIG-I)/RIP-1 pathways regulate the activation of IRFs and NF-κB in EBV-infected HCECs [80]. High levels of MMP-9 impair corneal epithelial function when EBV infection increases the levels of pro-inflammatory cytokines IL-6 and TNF-α and matrix metalloproteinases (MMP2 and MMP9). In addition, EBV-infected HCECs exhibit increased migratory and invasive capabilities compared with uninfected HCECs [81].

Punctual cautery increases tear meniscus height and alleviates epitheliopathy in acute DED [78]. Previous studies corroborate that acyclovir is effective in some cases of EBV-infection-associated ocular disease [82]. The topical application of steroids mitigates inflammation and oedema in the cornea [83]. Cyclosporin A (CsA) is thought to inhibit the activation of T lymphocytes and effectively control ocular inflammation in consequence [84].

### HCV

HCV is an RNA virus that belongs to the Flaviviridae family. HCV infection causes acute hepatitis C, and part of acute hepatitis C transforms into a chronic inflammatory disease process, which might lead to liver fibrosis, hepatocellular carcinoma, and terminal death [85]. HCV is primarily transmitted through percutaneous exposure to the blood caused by iatrogenic infections. Blood transfusion or the administration of clotting factors can cause iatrogenic infections.

HCV and DED have a direct causal relationship. The manifestations of chronic extrahepatic HCV infection include DED, which leads to tear film instability and ocular surface damage [86]. A study collects 36 tear samples from patients with dry eye, and 21 tear samples are positive for HCV RNA [86].

Patients with HCV infection have corneal inflammation, which contributes to DED. The HCV core is recognized by Toll-like receptors (TLR1, TLR2, and TLR6), and then the signal is transferred to myeloid differentiation factor 88 (MyD88), resulting in activation of the NF-κB pathway. HCV infection increases the protein levels of proinflammatory factors, including IL-6, IL-8, and TNF-α, in CECs and conjunctival fibroblasts. HCV core protein mediates nitric oxide (NO) production via the activation of inducible nitric oxide synthase (iNOS), facilitating the apoptosis of CECs and conjunctival fibroblasts [87,88]. The OSDI questionnaire, Schirmer I, TBUT, and ocular surface fluorescein were used to evaluate chronic hepatitis C (CHC) patients’ changes in ocular surface and tear function parameters in CHC patients. The results show that DED symptoms in CHC patients include reduced tear production, with increased OSDI and corneal straining scores [89].

Long-term artificial tears and topical use of Cyclosporin A in both eyes can cure the severe form of DED. Non-structural (NS) proteins, including NS3, NS5A, and NS5B, are required for HCV replication. Daclatasvir is an NS5A replication inhibitor that suppresses viral RNA synthesis and release [90]. Sofosbuvir is a nucleotide NS5B polymerase inhibitor. Combination medication with two or more direct-acting agents (DAAs) can effectively treat HCV infections. No intraocular complications were detected during the follow-up period of sofosbuvir ± daclatasvir ± ribavirin treatment for HCV-infected patients, indicating the safety of these treatments [91].

IFN is the drug of choice for treating HCV infection. After treatment with interferon α 2b and ribavirin for 16 weeks, serum HCV RNA decreased from the initial value to an undetectable level in HCV-infected patients [92]. IFN-free DAAs are widely used for the treatment of HCV. Schirmer’s test, TBUT, and PCR showed that IFN-free DAAs can improve ocular manifestations and reduce HCV RNA levels with sufficient safety [93].

## Potential therapeutic strategies for viral infection-associated dry eye

Evidence has shown that viral infections are associated with autoimmune disorders. SARS-CoV-2, HTLV-1, and EBV infections are associated with the sicca syndrome. HTLV-1 infection-related HSCT may contribute to severe complications such as chronic ocular graft-versus-host disease (GVHD) [94]. However, few direct drugs are available for curing viral infection-associated dry eye. Hydrocortisone combined with topical CsA can effectively treat Sjögren’s Syndrome [95]. As a well-known antiviral agent, type I IFN triggers an antiviral response within infected and target cells, as well as activates innate immune cells, which ultimately control viral replication, activate adaptive immune response to clear viral infection, and generate memory to create a rapid response against future infections [96]. The pathogenesis of DED includes inflammation, and keratitis can cause DED. Meanwhile, viral infection induces conjunctivitis and keratitis, as evidenced by a case report of a patient diagnosed with EBV retinitis [97]. Topical application of anti-inflammatory drugs can inhibit inflammatory mediators and relieve the symptoms and signs of DED. Traditional ocular topical anti-inflammatory drugs include tetracycline, glucocorticoids and nonsteroidal anti-inflammatory drugs. Rebamipide is an oral drug that increases the number of conjunctival goblet cells on the ocular surface. Furthermore, it suppresses T cell activation and cytokine production to exert anti-inflammatory effects [98]. Here, we summarize the treatment and effect of dry eye associated with viral infection (Table 1).

**Table 1.** The treatments and effects of DED associated with viral infection.

Virus	ocular surface disease	treatment drugs	therapeutic effect	References
SARS-CoV-2	DED	OED	Ameliorate the reduction of corneal epithelium thickness and inhibit viral replication.	[41]
SARS-CoV-2	DED	Ribavirin eye drop	Improve the ocular manifestations.	[99]
HIV	DED	cART	CD4 count decreased in ocular surface.	[56]
HIV	DED associated with DILS	artificial tear substitutes, autologous serum eye drops, anti-inflammatory agents	Reduce ocular surface inflammation and tear film instability	[48]
EBV	Acute DED	punctual cautery	Increase tear meniscus height. Improve the dryness and defect of ocular surface.	[78]
EBV	severe keratoconjunctivitis sicca	cyclosporin A and prednisone	Vision restoration and ocular inflammation reduction.	[100]
HCV	DED	DAAs treatment	Significantly improve the ocular manifestations. (Schirmer’s and TBUT values increase)	[93]
HTLV-1	DED	HSCT	Significantly improve eye complications. HTLV-1 PVL decreases.	[60]

OED, ozonated-oil in liposome eyedrop gel; cART, combined antiretroviral therapy; DILS, diffuse infiltrative lymphocytosis syndrome (a consequence of HIV infection); anti-inflammatory agents, corticosteroids, cyclosporine, lifitegrast; DAAs, direct-acting antiviral; HSCT, haematopoietic stem cell transplantation; PVL, proviral load.

## Conclusions and prospect

**Figure 1.**
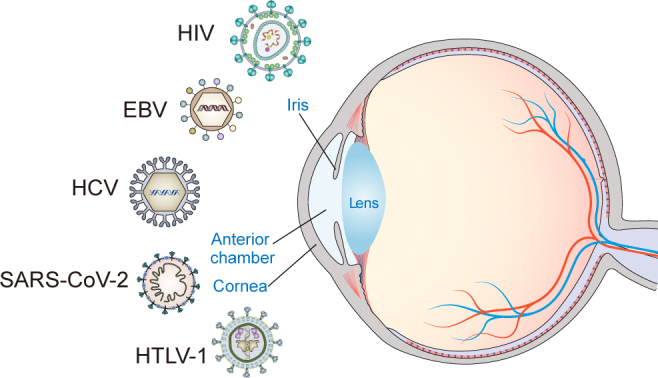
Different viruses, including HIV, EBV, HCV, SARS-CoV-2, and HTLV-1, infect eye surface.

**Figure 2.**
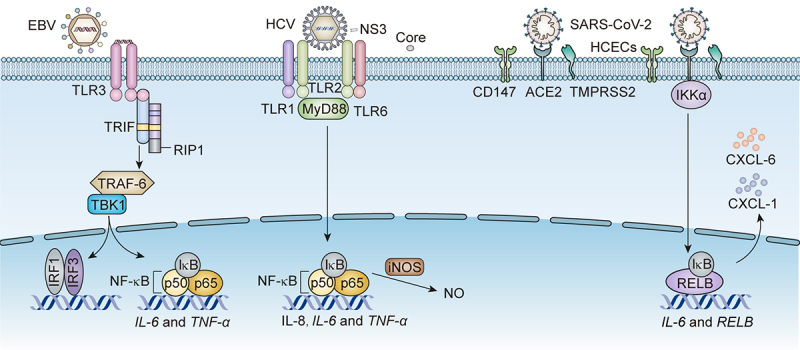
The receptors on CECs and downstream pathways for EBV, HCV, and SARS-CoV-2 infection-associated DED. EBV activates NF-κB to produce IL-6 and TNF-α via binding to TLR3 on CECs. Meanwhile, EBV promotes the transcription of IRF3 via potentiating the same upstream TRIF/RIP1/tumour necrosis factor receptor-associated factor 6 (TRAF-6)/TANK-binding kinase 1 (TBK1) signalling. The NS3 of HCV binds to TLR1 and TLR6 on CECs to activate NF-κB, producing IL-8, IL-6 and TNF-α via boosting upstream MyD88. The core protein of HCV enhances the generation of NO via activating iNOS. SARS-CoV-2 binds to CD147, ACE2, and TMPRSS2 on CECs, activating NF-κB pathway to produce IL-6, CXCL-1 and CXCL-6 through upstream inhibitor of kappa B kinase α (IKKα).

Multiple studies have revealed that systemic and ocular infections, as well as other environmental factors, may contribute to the development of DED. Similarly, many studies have confirmed the intimate relationship between viral infections and the ocular surface, lacrimal gland, and conjunctiva. A new multiplex solid-phase strip PCR can be used to identify common pathogens that cause ocular infectious diseases. Each well of the strip PCR targets one to three types of pathogens. The wells are coated with primers and probes containing the fluorescent reporter dye [6-carboxyfluorescein (6-FAM), hexachlorofluorescein (HEX), or cyanine-5 (Cy5)] on the 5’-end and the non-fluorescent quencher on the 3’-end [101]. Both strip PCR and capillary PCR have detected human herpesvirus 7 (HHV-7) in conjunctivitis and the tears of dry eye patients. Acanthamoeba, herpes simplex virus type 1 (HSV-1), bacterial 16S, human herpesvirus 6 (HHV-6), aspergillus, and fungal 28S were detected in the corneas of keratitis patients. Adenovirus, Propionibacterium acnes (P. acnes), and EBV were detected in the conjunctivas of infectious conjunctivitis patients [102]. A study indicated that interferon regulatory factor (irf3) ^−/−^ mice infected with HSV-1 display symptoms of DED, including corneal keratinization, corneal cell apoptosis, and tear production reduction. Irf3^−/−^ mice have pathological changes and functional impairment in the lacrimal glands (LGs) caused by increased levels of HSV-1 [103]. Here, we summarize the symptoms, causes, diagnosis, and treatment of viral infection-associated DED. A variety of viruses, including HIV, EBV, HCV, SARS-CoV-2, and HTLV-1, infect the ocular surface, and cause DED (Figure 1). We also generalized the receptors of EBV, HCV, and SARS-CoV-2 infecting CECs as well as the downstream pathways in DED associated with their infections (Figure 2). All three viruses activate the NF-κB pathway by binding to diverse receptors on the CECs. These findings raise the question of whether there are other mechanisms besides the NF-κB pathway that mediate EBV, HCV, and SARS-CoV-2 infections-associated DED. More importantly, the cellular and molecular mechanisms of HIV and HTLV-1 infection-associated DED remain unclear and require further investigation.

In this review, the importance of timely diagnosis of viral infections is emphasized. A deeper and wider understanding of the direct relationship between viral infections and DED is helpful for ophthalmologists to make accurate judgements in time and achieve effective treatments.

Dry eye symptoms in virus-infected patients, such as SARS-CoV-2, EBV, and HSV, have been reported. However, the relationship between other viruses, such as HSV, HTLV-1, HIV, and DED, and their symptoms and pathogenesis warrant further study. Here, we summarize DED symptoms in virus-infected patients and the routes of viral transmission (Table 2). Cell and animal models are urgently need to explore the pathogenesis of viral infection-associated DED. However, only a few animal models of viral infection-associated DED have been developed. Cell and animal models of viral infection-associated ocular surface diseases such as keratitis and keratouveitis have already been successfully constructed (Table 3). These models are beneficial for the investigation of viral infection-associated DED.

**Table 2.** The manifestations and routes of DED caused by viruses.

Virus	route of transmission	clinical manifestation/diagnostic results
SARS-CoV-2	Eye route [29], aerosols and/or droplets [19]	Dryness, discomfort of the eye [104]. Frequent and/or rapid blinking and pain of the eye [26], OSDI and tear film osmolarity increase. TBUT values decrease [105].
HIV	Transconjunctival exposure [106], ocular mucosal infection [107]	Inflammatory infiltration in LG [49]. Discomfort, foreign body sensation of the eye, TBUT reduction [56]. Meibomian gland dropout score, tear film osmolarity, and ocular surface staining increase [51].
HSV-1	Corneal infection [103]	Corneal keratinization, corneal cell apoptosis, tear reduction and inflammatory pathological changes and impaired function in LG [103].
EBV	Corneal epithelium infection [80]	Severe redness and photophobia in both eyes. Tear meniscus height reduction [78].
HTLV-1	Blood transfusion [108]	Dry eye accompanied with the decrease of Schirmer’s test values [63]. Red and pain of the eye, Schirmer values decrease. Fluorescein staining show defect in cornea [64].
HCV	Blood transfusions, intravenous drug use [109], ocular mucosal infection [107]	Tear volume and lactoferrin concentration decrease [86]. Burning and painful eyes, pruritus, epiphora, and a sensation of pressure of the eye. The goblet cell density reduction. Tear production decreases [109].
HBV	Ocular mucosal infection [107]	MG dropout score, meibum quality score and SPEED questionnaire score increases. NITBUT values decrease [110].

SPEED, standardized patient evaluation of eye dryness; NITBUT, non-invasive tear break-up time.

**Table 3.** The cell and animal models associated with viral infection.

Virus	Construction technique	mechanisms	ocular surface disease	References
HSV	4 μl 5 × 10^4^ virus in 2% DMEM was applied to irf3^−/−^ mouse cornea	Irf3 deficiency-activated PI3K/AKT pathway enhances HSV infection and destruction of LG and cornea	DED	[103]
HSV-1	3 μl PBS containing 1 × 10^5^ PFU HSV-1 was applied to scraped cornea of female C57BL/6J mouse	a massive influx of IFN-γ secreting T cells in EoLG induces reduced tear level and damaged tear film composition	HSK	[111]
HSV-1	5 μl solution containing 1 × 10^5^ PFU HSV-1 was applied to scraped cornea in male C57BL/6J mouse	HSV-1-induced Dot1l causes p38 MAPK up-regulation and oxidative stress in cornea	HSK	[112]
HAdVs	1 μl virus 10^5^ TCID_50_ was intrastromally applied to cornea of female C57BL/6J mouse	HAdVs induce the activation of TLR2, TLR9, and MyD88	Adenovirus Keratitis	[113]
MCMV	3 μl 60 PFU viral suspension was intracameral applied to cornea of female Sprague-Dawley rat	intraocular pressure increasing and inflammation of iris and ciliary body	CMVkeratouveitis	[114]
EBV	co-culture of HCECs and EBV-containing culture supernatant	The co-culture induces Syk and Src phosphorylation to activate PI3K/Akt and Erk which lead to EMT and migration of HCECs	keratitis	[81]
VACA	5 μl DMEM containing 1 × 10^7^ VACA was applied to scraped cornea of female C57BL/6J mouse	VACA induces the infiltration of neutrophils and lymphocytes in cornea	stromal keratitis	[115]
SARS-CoV-2	C57Bl/6 Ace2^−/−^ mouse	AACE2 deficiency activates AngII to produce excessive pro-inflammatory cytokines in cornea	blepharitis	[116]

Akt, protein kinase B; AngII, angiotensin II; Dot1l, disruptor of telomeric silencing 1-like; EMT, epithelial-mesenchymal transition; EoLG, extraorbital lacrimal gland; Erk, extracellular signal-regulated kinase; HAdVs, human adenoviruses; HCECs, human corneal epithelial cells; HSK, herpes stromal keratitis; HSV-1, herpes simplex virus 1; IFN-γ, interferon-gamma; MAPK, mitogen-activated protein kinase MCMV, mouse cytomegalovirus; MyD88, myeloid differentiation primary response protein 88; PFU, plaque forming units; PI3K, Phosphoinositide 3-kinase; Src, proto-oncogene tyrosine-protein kinase Src; Syk, spleen tyrosine kinase; TCID50, 50% tissue culture infective dose; VACA, vaccinia virus.

Currently, antiviral drugs (type I IFN and cART) are combined with drugs for dry eye (artificial tears and corticosteroids) and anti-inflammatory drugs, such as ADA and IFX. However, the current approaches to tackling DES remains suboptimal. Palliative medication using ophthalmic lubricants remains the backbone of DED treatment [117]. FDA-approved DED treatments, such as 5% lifitegrast ophthalmic solution (Xiidra®, Novartis, Switzerland) and 0.05% cyclosporine ophthalmic emulsion (Restasis®, Allergan, CA), show only finite efficacy for treating DED signs and symptoms, and are associated with related adverse events that hinder their widespread use in general patients globally [37,118]. Thus, it is pivotal to develop effective and safe DED treatments that target their authentic pathophysiology, which is a wicked cycle of desiccating stress-induced hyperosmolar tissue damage and inflammation of the surface [119,120]. Likewise, there is a need to investigate more drugs that target viral infection-associated DED.
